# Supplementary material for: Reliable long-term individual variation in wild chimpanzee technological efficiency
Source: Nat Hum Behav. 2024 Dec 23;9(3):472–80. doi: 10.1038/s41562-024-02071-8 (PMC11936830; doi:10.1038/s41562-024-02071-8)
Supplement: Supplementary file 1 — Supplementary Figs. 1–7, discussion and Tables 1–7. [file 41562_2024_2071_MOESM1_ESM.pdf]

nature human behaviour

Supplementary information https://doi.org/10.1038/s41562-024-02071-8

Reliable long-term individual variation in wild chimpanzee technological efficiency

In the format provided by the authors and unedited

Table of Contents

Individual variation ***1***
Figure S1. Stacked box plots of the intra-individual development of nut-cracking bout duration for each age class each chimpanzee was visible in the archive after their early learning period 1
Figure S2. Stacked box plots of the intra-individual development of strikes per nut for each age class each chimpanzee was visible in the archive after their early learning period 2
Figure S3. Bar chart of the intra-individual development of successfully extracting the whole oil palm kernel for each age class each chimpanzee was visible in the archive after their early learning period 3
Figure S4. Stacked forest plot of the intra-individual development of the number of nut displacements per for each age class each chimpanzee was visible in the archive after their early learning period 4
Figure S5. Stacked forest plot of the intra-individual development of the number of tool switches rate per bout for each age class each chimpanzee was visible in the archive after their early learning period 5
Reliability of individual variation ***6***
Figure S6. Correlation matrix for the Pearson's *r* correlation coefficient between the rankings for all pairs of nut-cracking efficiency measures. 6
Subject information ***7***
Table S1. Focal subject information, with the years that they were observably cracking nuts in the Bossou archive during their post-learning period. 7
Model outputs ***8***
Table S2. Simple and multilevel linear model results for log bout duration 8
Table S3. Simple and multilevel zero-truncated negative binomial model results for strikes per nut 9
Table S4. Simple and multilevel cumulative link model results for success rate 10
Table S5. Simple and multilevel zero-inflated negative binomial model results for displacement rate 11
Table S6. Simple and multilevel zero-inflated negative binomial model results for tool switch rate 12
Data collection protocol ***13***
Inter-rater reliability ***15***
Table S7. ICC calculations using single-rating, absolute agreement, two-way random-effects models 16
Assumption checks ***17***
Figure S7. The age predictor variable plotted against the model residuals 18
References ***19***

Individual variation

Figure S1. Stacked box plots of the intra-individual development of nut-cracking bout duration for each age class each chimpanzee was visible in the archive after their early learning period.

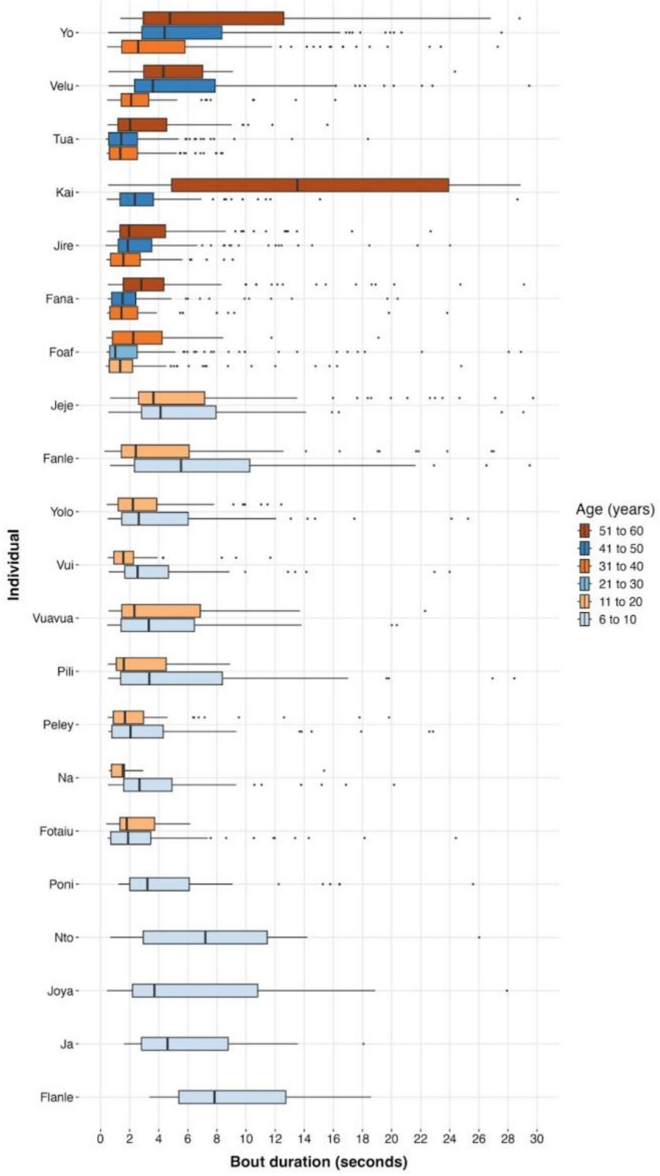

Boxplots show the lower quartile, median, and upper quartile of values for each age class for each individual. The whiskers show the minimum and maximum values, and dots show the outliers. Where the chimpanzee was in the video archive over multiple age classes, older ages are stacked on top of younger age classes. Sample sizes for each individual in each age class were as follows: Fana (31 to 40: *n* = 58, 41 to 50: *n* = 161, 51 to 60: *n* = 140), Fanle (6 to 10: *n* = 45, 11 to 20: *n* = 160), Flanle (6 to 10: *n* = 13), Foaf (11 to 20: *n* = 147, 21 to 30: *n* = 157, 31 to 40: *n* = 96), Fotaiu (6 to 10: *n* = 70, 11 to 20: *n* = 19), Ja (6 to 10: *n* = 19), Jeje (6 to 10: *n* = 37, 11 to 20: *n* = 140), Jire (31 to 40: *n* = 100, 41 to 50: *n* = 113, 51 to 60: *n* = 115), Joya (6 to 10: *n* = 20), Kai (41 to 50: *n* = 128, 51 to 60: *n* = 14), Na (6 to 10: *n* = 58, 11 to 20: *n* = 17), Nto (6 to 10: *n* = 9), Peley (6 to 10: *n* = 67, 11 to 20: *n* = 51), Pili (6 to 10: *n* = 55, 11 to 20: *n* = 36), Poni (6 to 10: *n* = 33), Tua (31 to 40: *n* = 106, 41 to 50: *n* = 169, 51 to 60: *n* = 49), Velu (31 to 40: *n* = 83, 41 to 50: *n* = 136, 51 to 60: *n* = 14), Vuavua (6 to 10: *n* = 61, 11 to 20: *n* = 34), Vui (6 to 10: *n* = 68, 11 to 20: *n* = 41), Yo (31 to 40: *n* = 144, 41 to 50: *n* = 145, 51 to 60: *n* = 46), and Yolo (6 to 10: *n* = 68, 11 to 20: *n* = 125).

Figure S2. Stacked box plots of the intra-individual development of strikes per nut for each age class each chimpanzee was visible in the archive after their early learning period.

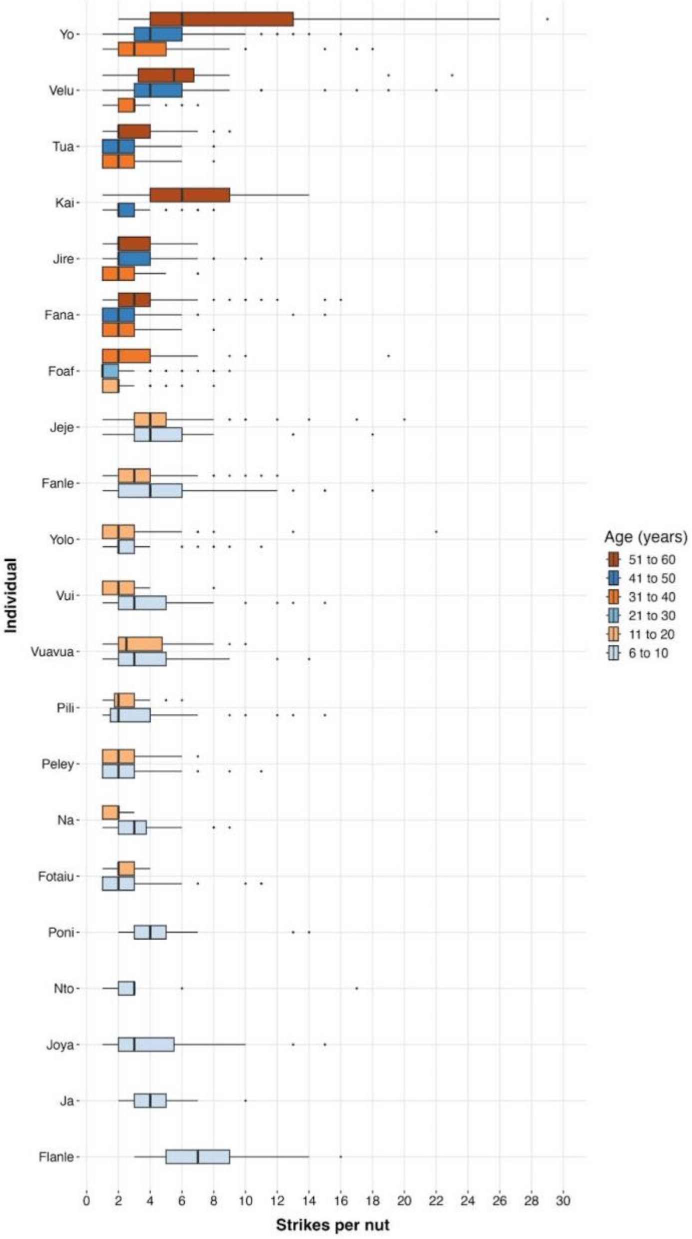

Boxplots show the lower quartile, median, and upper quartile of values for each age class for each individual. The whiskers show the minimum and maximum values, and dots show the outliers. Where the chimpanzee was in the video archive over multiple age classes, older ages are stacked on top of younger age classes. Sample sizes for each individual in each age class were as follows: Fana (31 to 40: *n* = 58, 41 to 50: *n* = 161, 51 to 60: *n* = 140), Fanle (6 to 10: *n* = 45, 11 to 20: *n* = 160), Flanle (6 to 10: *n* = 13), Foaf (11 to 20: *n* = 147, 21 to 30: *n* = 157, 31 to 40: *n* = 96), Fotaiu (6 to 10: *n* = 70, 11 to 20: *n* = 19), Ja (6 to 10: *n* = 19), Jeje (6 to 10: *n* = 37, 11 to 20: *n* = 140), Jire (31 to 40: *n* = 100, 41 to 50: *n* = 113, 51 to 60: *n* = 115), Joya (6 to 10: *n* = 20), Kai (41 to 50: *n* = 128, 51 to 60: *n* = 14), Na (6 to 10: *n* = 58, 11 to 20: *n* = 17), Nto (6 to 10: *n* = 9), Peley (6 to 10: *n* = 67, 11 to 20: *n* = 51), Pili (6 to 10: *n* = 55, 11 to 20: *n* = 36), Poni (6 to 10: *n* = 33), Tua (31 to 40: *n* = 106, 41 to 50: *n* = 169, 51 to 60: *n* = 49), Velu (31 to 40: *n* = 83, 41 to 50: *n* = 136, 51 to 60: *n* = 14), Vuavua (6 to 10: *n* = 61, 11 to 20: *n* = 34), Vui (6 to 10: *n* = 68, 11 to 20: *n* = 41), Yo (31 to 40: *n* = 144, 41 to 50: *n* = 145, 51 to 60: *n* = 46), and Yolo (6 to 10: *n* = 68, 11 to 20: *n* = 125).

Figure S3. Bar chart of the intra-individual development of successfully extracting the whole oil palm kernel for each age class each chimpanzee was visible in the archive after their early learning period.

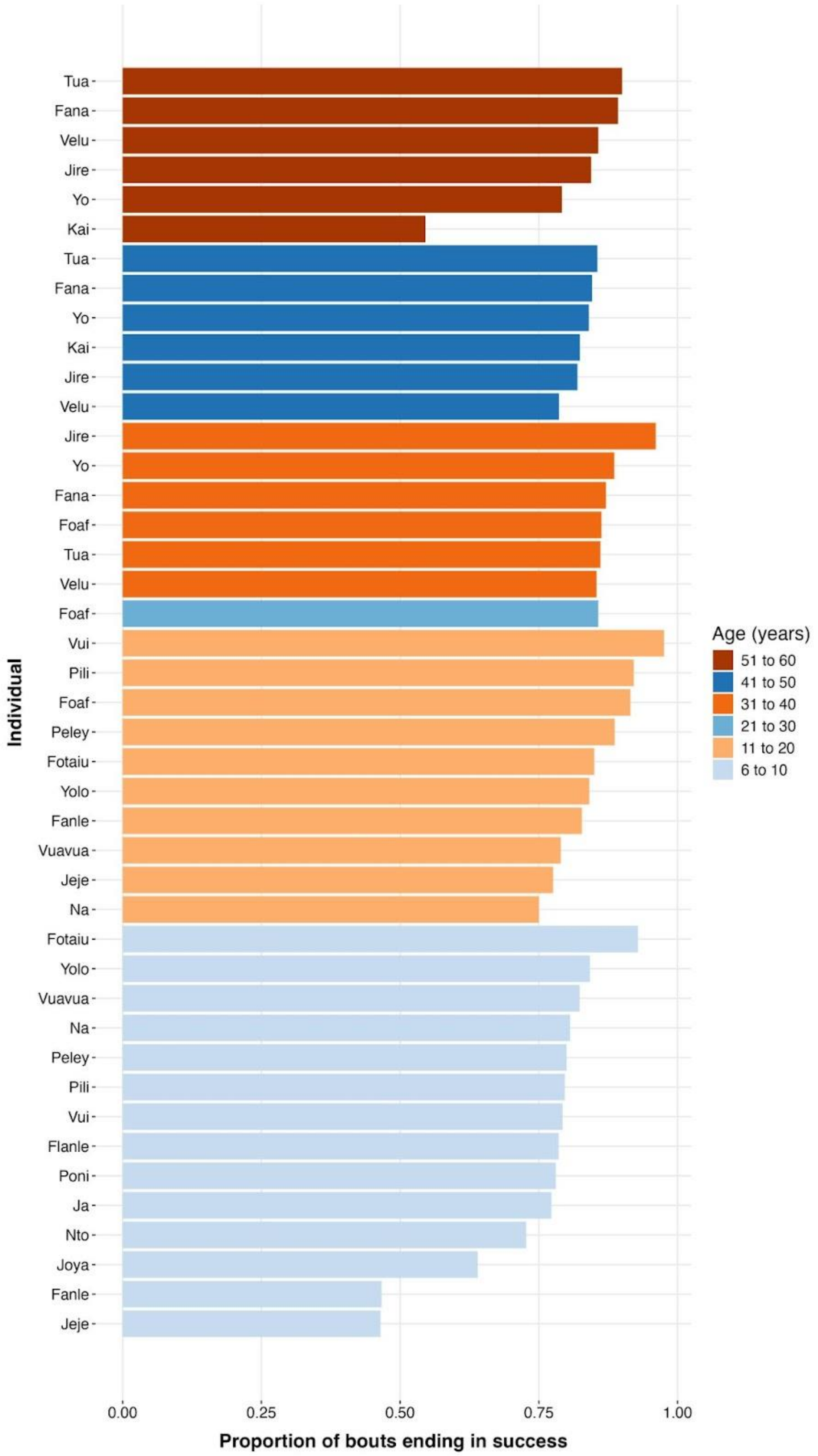

Figure S4. Stacked forest plot of the intra-individual development of the number of nut displacements per for each age class each chimpanzee was visible in the archive after their early learning period.

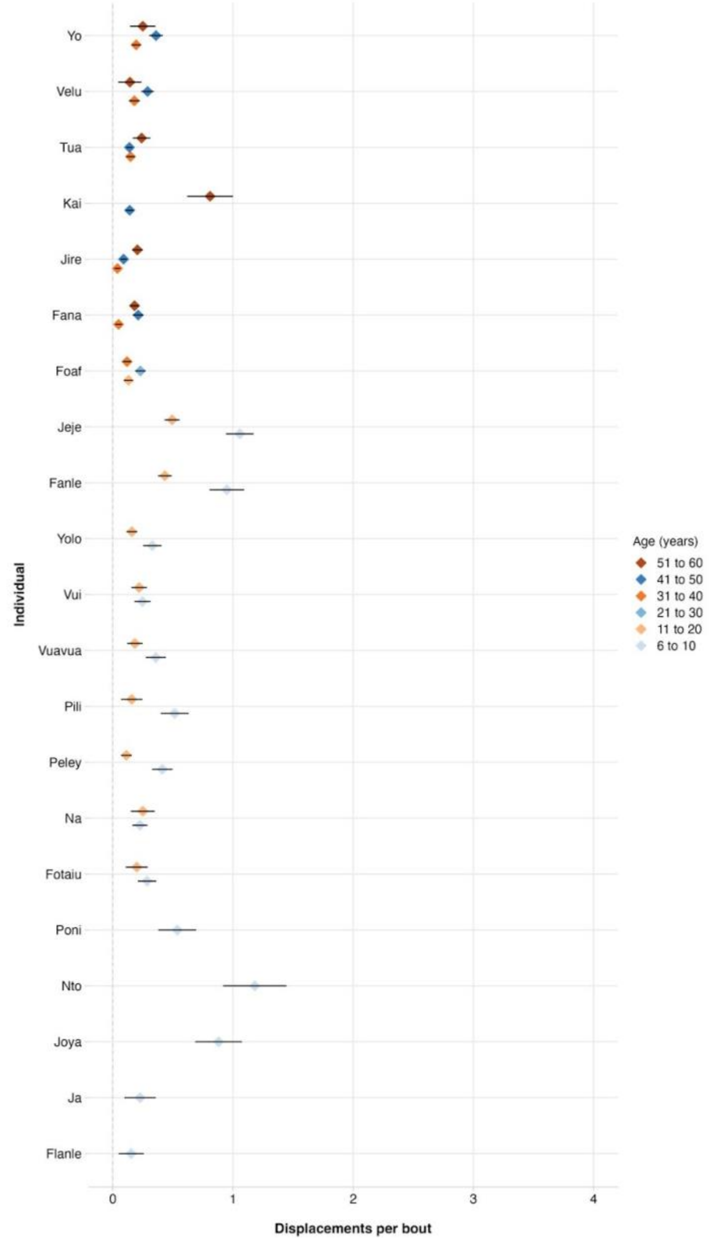

Diamonds represent the mean and the error bars represent the standard error of the mean. Where the chimpanzee was in the video archive over multiple age classes, older ages are stacked on top of younger age classes. Sample sizes for each individual in each age class were as follows: Fana (31 to 40: *n* = 61, 41 to 50: *n* = 175, 51 to 60: *n* = 149), Fanle (6 to 10: *n* = 60, 11 to 20: *n* = 174), Flanle (6 to 10: *n* = 14), Foaf (11 to 20: *n* = 153, 21 to 30: *n* = 161, 31 to 40: *n* = 102), Fotaiu (6 to 10: *n* = 70, 11 to 20: *n* = 20), Ja (6 to 10: *n* = 22), Jeje (6 to 10: *n* = 71, 11 to 20: *n* = 165), Jire (31 to 40: *n* = 102, 41 to 50: *n* = 122, 51 to 60: *n* = 122), Joya (6 to 10: *n* = 25), Kai (41 to 50: *n* = 142, 51 to 60: *n* = 22), Na (6 to 10: *n* = 62, 11 to 20: *n* = 20), Nto (6 to 10: *n* = 11), Peley (6 to 10: *n* = 80, 11 to 20: *n* = 53), Pili (6 to 10: *n* = 64, 11 to 20: *n* = 38), Poni (6 to 10: *n* = 41), Tua (31 to 40: *n* = 115, 41 to 50: *n* = 173, 51 to 60: *n* = 50), Velu (31 to 40: *n* = 89, 41 to 50: *n* = 145, 51 to 60: *n* = 14), Vuavua (6 to 10: *n* = 68, 11 to 20: *n* = 38), Vui (6 to 10: *n* = 82, 11 to 20: *n* = 41), Yo (31 to 40: *n* = 149, 41 to 50: *n* = 150, 51 to 60: *n* = 48), and Yolo (6 to 10: *n* = 76, 11 to 20: *n* = 132).

Figure S5. Stacked forest plot of the intra-individual development of the number of tool switches rate per bout for each age class each chimpanzee was visible in the archive after their early learning period.

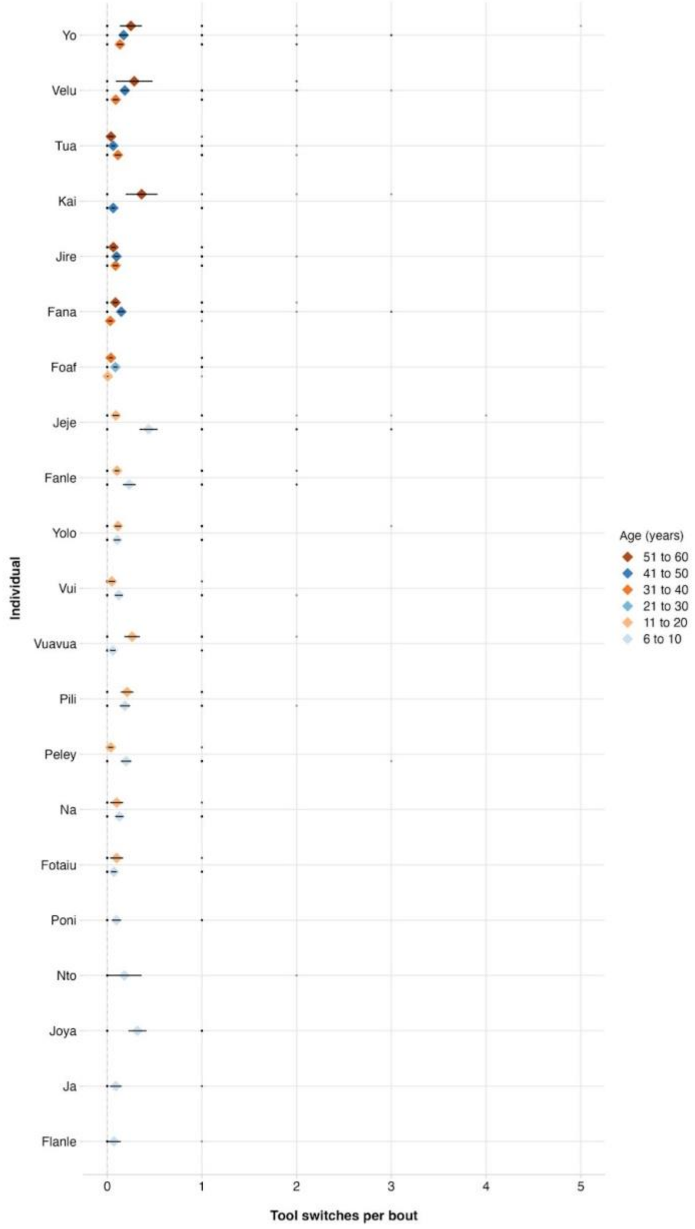

Diamonds represent the mean and the error bars represent the standard error of the mean. Where the chimpanzee was in the video archive over multiple age classes, older ages are stacked on top of younger age classes. Sample sizes for each individual in each age class were as follows: Fana (31 to 40: *n* = 61, 41 to 50: *n* = 175, 51 to 60: *n* = 149), Fanle (6 to 10: *n* = 60, 11 to 20: *n* = 174), Flanle (6 to 10: *n* = 14), Foaf (11 to 20: *n* = 153, 21 to 30: *n* = 161, 31 to 40: *n* = 102), Fotaiu (6 to 10: *n* = 70, 11 to 20: *n* = 20), Ja (6 to 10: *n* = 22), Jeje (6 to 10: *n* = 71, 11 to 20: *n* = 165), Jire (31 to 40: *n* = 102, 41 to 50: *n* = 122, 51 to 60: *n* = 122), Joya (6 to 10: *n* = 25), Kai (41 to 50: *n* = 142, 51 to 60: *n* = 22), Na (6 to 10: *n* = 62, 11 to 20: *n* = 20), Nto (6 to 10: *n* = 11), Peley (6 to 10: *n* = 80, 11 to 20: *n* = 53), Pili (6 to 10: *n* = 64, 11 to 20: *n* = 38), Poni (6 to 10: *n* = 41), Tua (31 to 40: *n* = 115, 41 to 50: *n* = 173, 51 to 60: *n* = 50), Velu (31 to 40: *n* = 89, 41 to 50: *n* = 145, 51 to 60: *n* = 14), Vuavua (6 to 10: *n* = 68, 11 to 20: *n* = 38), Vui (6 to 10: *n* = 82, 11 to 20: *n* = 41), Yo (31 to 40: *n* = 149, 41 to 50: *n* = 150, 51 to 60: *n* = 48), and Yolo (6 to 10: *n* = 76, 11 to 20: *n* = 132).

Reliability of individual variation

Figure S6. Correlation matrix for the Pearson's *r* correlation coefficient between the rankings for all pairs of nut-cracking efficiency measures.

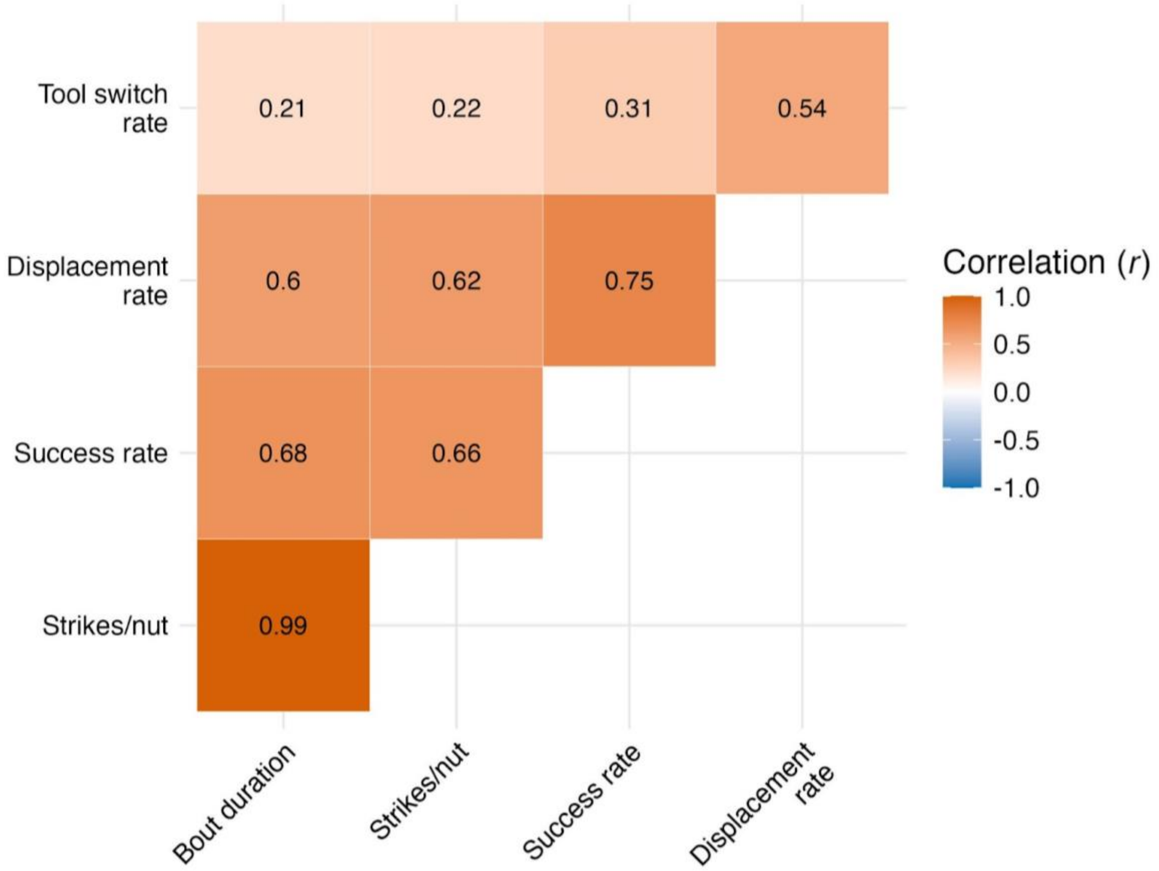

Subject information

Table S1. Focal subject information, with the years that they were observably cracking nuts in the Bossou archive during their post-learning period.

Subject	Sex	Nut-cracking hand	Observation years	Age (years)	Bouts observed
Fana	F	Right*	1992–2017	36–60	386
Jire	F	Left	1992–2017	34–59	346
Yo	F	Left	1992–2016	32–56	347
Tua	M	Left	1992–2012	35–55	338
Velu	F	Right	1992–2015	33–55	248
Kai	F	Right	1992–2002	42–52	164
Foaf	M	Right	1992–2017	12–37	416
Fanle	F	Right	2004–2017	6–20	234
Jeje	M	Left	2004–2017	7–20	236
Yolo	M	Left	1998–2009	6–17	208
Peley	M	Left	2005–2012	6–14	133
Pili	F	Right	1993–2000	6–13	102
Vui	M	Left	1992–1999	6–13	123
Vuavua	F	Left	1998–2004	6–12	106
Fotaiu	F	Right	1998–2003	6–11	90
Na	M	Right	1992–1996	7–11	82
Ja	F	Right	1992–1993	9–10	22
Poni	M	Right	2000–2002	7–9	41
Joya	F	Left	2010–2012	6–8	25
Flanle	M	Left	2014	6	14
Nto	F	Right	2000	6	11

* = Switched to her right hand after her left arm became paralysed in 1996.

Model outputs

Table S2. Simple and multilevel linear model results for log bout duration.

	**Simple model**			**Multilevel model**		
Predictors	*Estimates*	*CI*	*p*	*Estimates*	*CI*	*p*
(Intercept)	1.21	1.11–1.30	<0.001***	0.59	0.18–1.01	0.005**
Age	-0.01	-0.01–-0.00	<0.001***	0.02	0.02–0.03	<0.001***
Sex [Male]	-0.35	-0.42–-0.27	<0.001***	0.07	-0.53–0.67	0.812
**Random Effects**						
σ^2				0.94		
τ_{00}				0.46_{Subject}		
ICC				0.33		
N				21_{Subject}		
Observations	3367			3367		
R^2/R^2 adjusted	0.023 / 0.022			0.086 / 0.387		

Simple linear model and multilevel linear model outputs. The statistical tests were two-tailed. The confidence intervals are calculated using the standard error for the fixed effects. The random effects residual variance (σ^2) and intercept variance (τ_{00}) are presented. *$P < 0.05$; **$P < 0.01$; ***$P < 0.001$.

Table S3. Simple and multilevel zero-truncated negative binomial model results for strikes per nut.

	**Simple model**			**Multilevel model**		
Predictors	*Incidence Rate Ratios*	*CI*	*p*	*Incidence Rate Ratios*	*CI*	*p*
(Intercept)	3.04	2.76–3.34	<0.001***	1.68	1.14–2.47	0.008**
age	1.00	1.00–1.00	0.268	1.02	1.02–1.03	<0.001***
sex [Male]	0.71	0.66–0.77	<0.001***	1.12	0.65–1.94	0.684
**Random Effects**						
σ^2				0.49		
τ_{00}				0.39_{Subject}		
ICC				0.44		
N				21_{Subject}		
Observations	3367			3367		
R^2 conditional/ R^2 marginal	NA / 0.028			0.150 / 0.525		

Simple zero-truncated negative binomial model and multilevel zero-truncated negative binomial model outputs. The statistical tests were two-tailed. The confidence intervals are calculated using the standard error for the fixed effects. The random effects residual variance (σ^2) and intercept variance (τ_{00}) are presented. *$P < 0.05$; **$P < 0.01$; ***$P < 0.001$.

Table S4. Simple and multilevel cumulative link model results for success rate.

	**Simple model**			**Multilevel model**		
Predictors	*Odds Ratios*	*CI*	*p*	*Odds Ratios*	*CI*	*p*
Failed|Smash	0.09	0.08–0.10	<0.001***	0.09	0.07–0.12	<0.001***
Smash|Successful	0.20	0.18–0.23	<0.001***	0.21	0.17–0.27	<0.001***
Sex [Male]	0.96	0.81–1.14	0.641	0.98	0.67–1.43	0.897
N				21_{Subject}		
Observations	3672			3672		
R^2 Nagelkerke	0.000			NA		

Cumulative link model and cumulative link multilevel model outputs. The statistical tests were two-tailed. *$P < 0.05$; **$P < 0.01$; ***$P < 0.001$.

Table S5. Simple and multilevel zero-inflated negative binomial model results for displacement rate.

	Simple model			Multilevel model		
Predictors	*Incidence Rate Ratios*	*CI*	*p*	*Incidence Rate Ratios*	*CI*	*p*
(Intercept)	0.56	0.47–0.65	<0.001***	0.43	0.30–0.62	<0.001***
(Intercept)	0.56	0.47–0.65	<0.001***	1.51	1.38–1.69	
age	0.98	0.98–0.98	<0.001***	0.99	0.98–1.00	0.016*
sex [Male]	0.79	0.68–0.92	0.002**	0.81	0.54–1.21	0.303
(Intercept)	1.60	1.45–1.80		0.43	0.30–0.62	<0.001***
(Intercept)	1.60	1.45–1.80		1.51	1.38–1.69	
**Zero-Inflated Model**						
(Intercept)	0.00	0.00–Inf	0.994	0.00	0.00–Inf	0.993
**Random Effects**						
σ^2				1.68		
τ_{00}				0.16_{Subject}		
ICC				0.09		
N				21_{Subject}		
Observations	3672			3672		
R^2 conditional / R^2 marginal	NA / 0.056			0.020 / 0.104		

Simple zero-inflated negative binomial model and multilevel zero-inflated negative binomial model outputs. The statistical tests were two-tailed. The confidence intervals are calculated using the standard error for the fixed effects. The random effects residual variance (σ^2) and intercept variance (τ_{00}) are presented. *$P < 0.05$; **$P < 0.01$; ***$P < 0.001$.

Table S6. Simple and multilevel zero-inflated negative binomial model results for tool switch rate.

	**Simple model**			**Multilevel model**		
Predictors	*Incidence Rate Ratios*	*CI*	*p*	*Incidence Rate Ratios*	*CI*	*p*
(Intercept)	0.19	0.15–0.24	<0.001***	0.18	0.14–0.25	<0.001***
(Intercept)	0.19	0.15–0.24	<0.001***	1.32	1.21–1.50	
age	0.99	0.98–1.00	0.002**	0.99	0.98–1.00	0.014*
sex [Male]	0.66	0.53–0.83	<0.001***	0.68	0.50–0.92	0.012*
(Intercept)	1.33	1.22–1.51		0.18	0.14–0.25	<0.001***
(Intercept)	1.33	1.22–1.51		1.32	1.21–1.50	
**Zero-Inflated Model**						
(Intercept)	0.00	0.00–Inf	0.995	0.00	0.00–Inf	0.995
**Random Effects**						
σ^2				2.43		
τ_{00}				0.04_{Subject}		
ICC				0.02		
N				21_{Subject}		
Observations	3672			3672		
R^2 conditional / R^2 marginal	NA / 0.017			0.016 / 0.031		

Simple zero-inflated negative binomial model and multilevel zero-inflated negative binomial model outputs. The statistical tests were two-tailed. The confidence intervals are calculated using the standard error for the fixed effects. The random effects residual variance (σ^2) and intercept variance (τ_{00}) are presented. *$P < 0.05$; **$P < 0.01$; ***$P < 0.001$.

Data collection protocol

First, all available footage was systematically reviewed to ascertain the party composition in each video, and verify which chimpanzees were visible cracking nuts in the footage. Following this, the videos' unique identifiers (UIDs) for each year were placed into ascending order and allocated an integer code starting from one, incrementally increasing by one. Vectors for each focal individual in each year were constructed in RMarkdown[1], each comprising the code for each video the individual was nut-cracking. Each vector was sampled without replacement to create a random order of video codes for all individuals. A seed was set to make the sequence of random codes replicable.

From this process, it became apparent that some individuals in the community (for example, Velu and Fana) were present and nut-cracking in the footage considerably less frequently than the other community members. To reduce potential bias introduced from the varying sample sizes for each subject, data from all nut-cracking bouts for the rare individuals (defined as being present and having observable nut-cracking bouts in ≤ 25% of videos for a given year) were collected. Where other chimpanzees had observable nut-cracking bouts in this footage, data from their bouts were also collected. This allowed for the effects of seasonality on nut hardness to be partially controlled. Thereafter, the videos were selected from the randomly ordered vectors (present and nut-cracking in > 25% of videos for a given year), starting from the least common of the remaining chimpanzees. This process continued until at least 20 nut-cracking bouts had been recorded for each individual.

Data from each year each individual was present in the archive was collected. Multiple bouts per individual per year were recorded to establish the degree of within-individual variation in efficiency, while also producing more independent data points, allowing between-individual variation to be assessed. This reduced the sampling error and random variation found between

years, and hence amplified the signal-to-noise ratio. This was to ensure that the data collected were reliable, and representative of the true behaviour of the group.

Lastly, to ensure the measures of efficiency were recorded accurately, only bouts which were clearly visible (i.e., observable) were coded. Visible bouts were those where the focal individual was facing the camera and the nut, hammer, and anvil could be seen, and those where the individual was not directly facing the camera, but the nut, hammer, and anvil could be seen. At the end of each bout, whether or not the complete bout was observed was recorded. Incomplete bouts were removed prior to analysis. This reduced the risk of systematic bias being introduced into the sampling procedure by the recording period ending prior to the termination of the behaviour, or because the focal subject became occluded[2].

Inter-rater reliability

Two independent, hypothesis-blind coders were recruited to test the between-observer reliability of the five efficiency components. This took place following the pilot research to ensure that 1) the coding scheme was finalised prior to the main data collection, and 2) that the coding scheme was consistent throughout the investigation, as any ambiguities in the behavioural category definitions were clarified *a priori*. This was important for reducing potential disagreement, and hence increasing reliability, between coders.

Both independent coders received thorough training for using the coding scheme and the BORIS software. Thereafter there was no consultation between coders, although the identity of the individuals in the videos were provided to assist with the accuracy of the behavioural coding.

The videos for reliability analysis were randomly selected to reduce the risk of bias. A combined total of 70 hours of observation was completed by the independent coders.

Cohen's κ was calculated to determine the extent of agreement between coders for *success rate* as the measure was categorical. All statements of the strength of the agreement between the coders are in accordance with standardised benchmarks[3].

Numerical data were compared using intraclass correlations (ICCs). Here, two-way random-effects models were used. The type was selected to be 'single rater' since measurements were not averaged across the *k* number of raters. Finally, 'definition' varies depending on the variable. *Strikes per nut*, *displacement rate*, and *tool switch rate* were selected as 'absolute agreement' to check if scores matched exactly across coders. *Bout duration* was selected as 'consistency' to determine the extent to allow for systematic error[4]. All statements of the strength of the agreement between the coders are in accordance with standardised guidelines[4]. Analyses were performed using the *irr* package[5]. ICC scores to assess the absolute agreement between the three raters can be found in Table S7. For *bout outcome*, the agreement between

the three coders was substantial, κ = 0.771, and greater than what would be expected by chance, Z = 19.5, p <0.0001. For *bout duration*, a single-rater, consistency, two-way model ICC analysis found F(424,424) = 214, p < 0.0001, ICC(C,1) = 0.991, 0.989 < ICC < 0.992, indicating excellent consistency.

Table S7. ICC calculations using single-rating, absolute agreement, two-way random-effects models.

	ICC	95% Confidence Interval		F Test with True Value 0			
		Lower bound	Upper bound	Value	*df1*	*df*2	*p*
Strikes per nut	0.986	0.984	0.989	147	424	424	<0.001***
Displacement rate	0.893	0.872	0.91	17.6	424	424	<0.001***
Tool switch rate	0.708	0.652	0.755	6.03	424	305	<0.001***

**P* < 0.05; ***P* < 0.01; ****P* < 0.001.

Assumption checks

After fitting the models, the assumptions were checked to ensure inferences could be drawn from the findings. Multicollinearity between predictor variables for the log *bout duration* model was checked using the *vif* function (variance inflation factor; VIF) in the *car* package[6]. The VIF was 1.13, indicating no issues of multicollinearity.

For the linear multilevel model (log *bout duration*), the normality of the residuals was assessed visually using QQ-plots and homoscedasticity was checked by plotting the fitted values against the squared residuals[7]. For the cumulative link model (*success rate*), surrogate residuals[8] were obtained using the *sure* package[9]. We performed assumption checks on the simple model (CLM) as the package does not currently support multilevel models (CLMM). We assumed this would be sufficient as only the intercepts were allowed to vary in the CLMM. The normality of the surrogate residuals was assessed visually using a QQ-plot and homoscedasticity was checked by plotting the fitted values against the surrogate residuals[10]. For all models, the normality of the random intercepts were assessed using QQ-plots and Shapiro-Wilk tests. Results indicated no significant deviations from normality.

For the four models with age included as a fixed effect (log *bout duration*, *strikes per nut*, *displacement rate*, *tool switch rate*), we checked the assumption that age was linearly related to the outcome measure by plotting the model residuals against the predictor variable. We found that age appears to be linearly related to our outcome variables (see Figure S7), and as such we did not need to square the age term in the models.

Figure S7. The age predictor variable plotted against the model residuals.

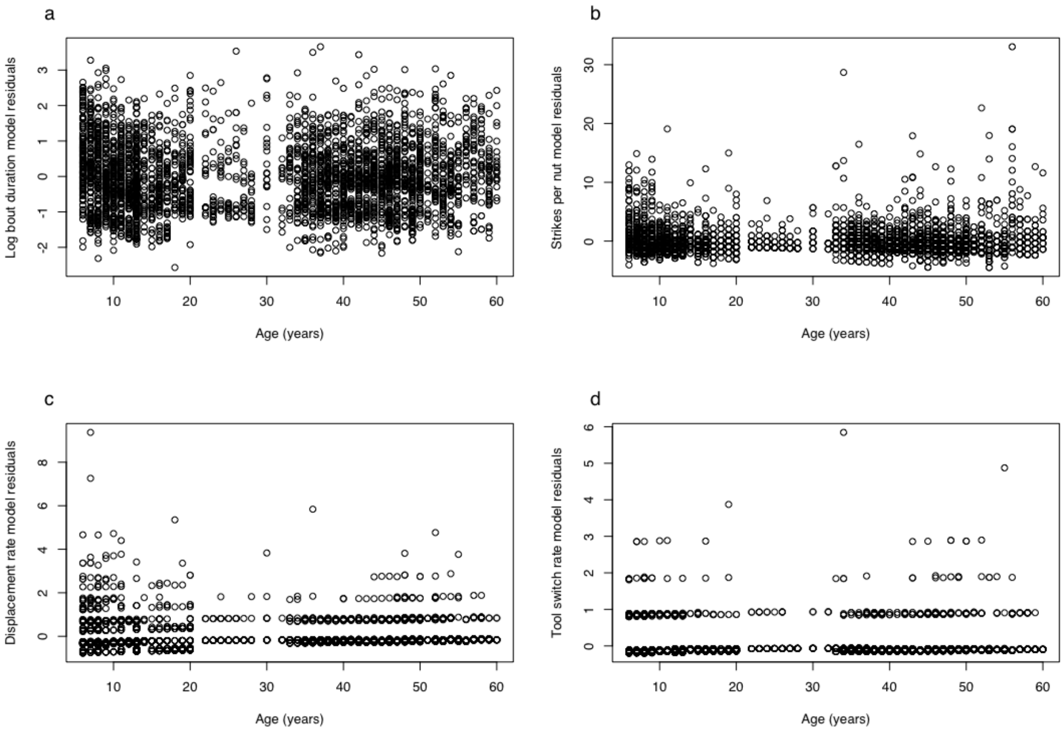

Age plotted against the model residuals for **a,** log bout duration, **b,** strikes per nut, **c,** displacement rate, and **d,** tool switch rate. Age is from 6–60 years old.

We evaluated the multilevel models using influence diagnostics from the *influence.ME* package[11]. DFBETA values were calculated for each model to assess whether any individuals (i.e., the random effect) had an outsized influence on the results of the models. For log *bout duration*, two individuals (Fana and Yo) had DFBETA values above the $2/\sqrt{n}$ cut-off[12], indicating that their data were influential. We re-ran the multilevel model (with individual as a random intercept, and age and sex as fixed effects) excluding their data and compared it to a simple linear model (without random effects). The random intercept model fit the data significantly better than the simple model ($\chi^2(1) = 203.75$, $p < 0.0001$), and as such we kept their data included in the model. We found no influential individuals in the *strikes per nut, displacement rate,* and *tool switch rate* models.

References

1. Allaire, J. J. *et al.* rmarkdown: dynamic documents for R. (2022).
2. Bateson, M. & Martin, P. *Measuring behaviour: an introductory guide*. (Cambridge University Press, 2021).
3. Landis, J. R. & Koch, G. G. The measurement of observer agreement for categorical data. *Biometrics* **33**, 159–174 (1977).
4. Koo, T. K. & Li, M. Y. A guideline of selecting and reporting intraclass correlation coefficients for reliability research. *J. Chiropr. Med.* **15**, 155–163 (2016).
5. Gamer, M., Lemon, J., Fellows, I. & Singh, P. irr: various coefficients of interrater reliability and agreement. (2019).
6. Fox, J. & Weisberg, S. *An R companion to applied regression*. (Sage, 2019).
7. Tkaczynski, P. *et al.* Long-term repeatability in social behaviour suggests stable social phenotypes in wild chimpanzees. *R. Soc. Open Sci.* **7**, 200454 (2020).
8. Liu, D. & Zhang, H. Residuals and diagnostics for ordinal regression models: a surrogate approach. *J. Am. Stat. Assoc.* **113**, 845–854 (2018).
9. Greenwell, B., McCarthy, A. & Boehmke, B. sure: surrogate residuals for ordinal and general regression models. (2017).
10. Greenwell, B., McCarthy, A., Boehmke, B. C. & Liu, D. Residuals and diagnostics for binary and ordinal regression models: an introduction to the sure package. *R J.* **10**, 381 (2018).
11. Nieuwenhuis, R., Te Grotenhuis, H. F. & Pelzer, B. J. Influence.ME: tools for detecting influential data in mixed effects models. *R J.* **4**, 38–47 (2012).
12. Belsley, D. D., Kuh, E. & Welsch, R. E. *Regression diagnostics: identifying influential data and sources of collinearity*. (Wiley, 1980).
